# Supplementary material for: Mutation Patterns of Human SARS-CoV-2 and Bat RaTG13 Coronavirus Genomes Are Strongly Biased Towards C>U Transitions, Indicating Rapid Evolution in Their Hosts
Source: Genes (Basel). 2020 Jul 7;11(7):761. doi: 10.3390/genes11070761 (PMC7397057; doi:10.3390/genes11070761)
Supplement: Supplementary file 1 [file genes-11-00761-s001.zip › Table S5.docx]

**Table S5.** Nucleotide substitutions and their characteristics for 33 SARS-CoV-2 isolates compared to the reference strain MN908947.

| **Position (genome)^1^** | **Base change** | **Position (mature peptide)^2^** | **Type of mutation^3^** | **Hydrophobicity^4^** | | | **Region^7^** |
| --- | --- | --- | --- | --- | --- | --- | --- |
|  |  |  |  | **Ref.^5^** | **Allele^5^** | **Shift^6^** |  |
| 75 | CA |  | nc |  |  |  | 5'UTR |
| 241 | CU |  | nc |  |  |  | 5'UTR |
| 490 | UA | Asp75Glu | S | -3.5 | -3.5 | 0 | ORF1ab-nsp1 |
| 565 | UC | Ser100Ser | S | -0.8 | -0.8 | 0 | ORF1ab-nsp1 |
| 884 | CU | Arg27Cys | NS | -4.5 | 2.5 | 7 | ORF1ab-nsp2 |
| 1059 | CU | Thr85Ile | NS | -0.7 | 4.5 | 5.2 | ORF1ab-nsp2 |
| 1348 | CU | Pro180Pro | S | -1.6 | -1.6 | 0 | ORF1ab-nsp2 |
| 1397 | GA | Val198Ile | NS | 4.2 | 4.5 | 0.3 | ORF1ab-nsp2 |
| 2269 | AU | Ala488Ala | S | 1.8 | 1.8 | 0 | ORF1ab-nsp2 |
| 2277 | UC | Ile491Thr | NS | 4.5 | -0.7 | -5.2 | ORF1ab-nsp2 |
| 2717 | GA | Gly818Ser | NS | -0.4 | -0.8 | -0.4 | ORF1ab-nsp3 |
| 2971 | GU | Met84Ile | NS | 1.9 | 4.5 | 2.6 | ORF1ab-nsp3 |
| 3037 | CU | Phe106Phe | S | 2.8 | 2.8 | 0 | ORF1ab-nsp3 |
| 3177 | CU | Pro153Leu | NS | -1.6 | 3.8 | 5.4 | ORF1ab-nsp3 |
| 3373 | CA | Asp218Glu | NS | -3.5 | -3.5 | 0 | ORF1ab-nsp3 |
| 3518 | GU | Leu37Phe | NS | 3.8 | 2.8 | -1 | ORF1ab-nsp3 |
| 3778 | AG | Thr353Thr | S | -0.7 | -0.7 | 0 | ORF1ab-nsp3 |
| 4402 | UC | Leu561Leu | S | 3.8 | 3.8 | 0 | ORF1ab-nsp3 |
| 5062 | GU | Leu37Phe | NS | 3.8 | 2.8 | -1 | ORF1ab-nsp3 |
| 6031 | CU | Asn1104Asn | S | -3.5 | -3.5 | 0 | ORF1ab-nsp3 |
| 6695 | CU | Pro1326Ser | NS | -1.6 | -0.8 | 0.8 | ORF1ab-nsp3 |
| 8388 | AG | Lys1890Arg | NS | -4.5 | -3.9 | 0.8 | ORF1ab-nsp3 |
| 8782 | CU | Ser76Ser | S | -0.8 | -0.8 | 0 | ORF1ab-nsp4 |
| 8945 | AG | Asn131Arg | NS | -3.5 | -4.5 | -1 | ORF1ab-nsp4 |
| 8987 | UA | Phe145Ile | NS | 2.8 | 4.5 | 1.7 | ORF1ab-nsp4 |
| 9159 | CU | Pro202Leu | NS | -1.6 | 3.8 | 5.4 | ORF1ab-nsp4 |
| 9274 | AG | Arg240Arg | S | -4.5 | -4.5 | 0 | ORF1ab-nsp4 |
| 9477 | UA | Phe308Tyr | NS | 2.8 | -1.3 | -4.1 | ORF1ab-nsp4 |
| 10232 | CU | Arg60Cys | NS | -4.5 | 2.5 | 7 | ORF1ab nsp5 |
| 11083 | GU | Leu37Phe | NS | 3.8 | 2.8 | -1 | ORF1ab-nsp6 |
| 11083 | GC | Leu37Phe | NS | 3.8 | 2.8 | -1 | ORF1ab-nsp6 |
| 12115 | CU | Ser8Ser | S | -0.8 | -0.8 | 0 | ORF1ab-nsp8 |
| 13225 | CG | Ser67Ser | S | -0.8 | -0.8 | 0 | ORF1ab-nsp10 |
| 13226 | UC | Phe69Leu | NS | 2.8 | 3.8 | 1 | ORF1ab-nsp10 |
| 14408 | CU | Pro323Leu | NS | -1.6 | 3.8 | 5.4 | ORF1ab-nsp12 |
| 14657 | CU | Leu406Phe | NS | 3.8 | 2.8 | -1 | ORF1ab-nsp12 |
| 14805 | CU | Thr454Ile | NS | -0.7 | 4.5 | 5.2 | ORF1ab-nsp12 |
| 15597 | UC | Met719Thr | NS | 1.9 | -0.7 | -2.6 | ORF1ab-nsp12 |
| 17247 | UC | Arg337Arg | S | -4.5 | -4.5 | 0 | ORF1ab-nsp13 |
| 17373 | CU | Ala379Ala | S | 1.8 | 1.8 | 0 | ORF1ab-nsp13 |
| 17376 | AG | Thr380Thr | S | -0.7 | -0.7 | 0 | ORF1ab-nsp13 |
| 17423 | AG | Tyr396Cys | NS | -1.3 | 2.5 | 3.8 | ORF1ab-nsp13 |
| 17747 | CU | Pro504Leu | NS | -1.6 | 3.8 | 5.4 | ORF1ab-nsp13 |
| 17825 | CU | Thr530Ile | NS | -0.7 | 4.5 | 5.2 | ORF1ab-nsp13 |
| 17858 | AG | Tyr541Cys | NS | -1.3 | 2.5 | 3.8 | ORF1ab-nsp13 |
| 18060 | CU | Leu7Leu | S | 3.8 | 3.8 | 0 | ORF1ab-nsp14 |
| 19065 | UC | Pro342Pro | S | -1.6 | -1.6 | 0 | ORF1ab-nsp14 |
| 19839 | UC | Asn73Asn | S | -3.5 | -3.5 | 0 | ORF1ab-nsp15 |
| 20936 | CU | Thr93Met | NS | -0.7 | 1.9 | 2.6 | ORF1ab -nsp16 |
| 21644 | UA | Tyr28Lys | NS | -1.3 | -3.9 | -2.6 | S-protein |
| 22224 | CG | Ser221Trp | NS | -0.8 | -0.9 | -0.1 | S-protein |
| 22785 | GU | Arg408Ile | NS | -4.5 | 4.5 | 9 | S-protein |
| 22984 | GA | Gln474Gln | S | -3.5 | -3.5 | 0 | S-protein |
| 22303 | UG | Ser247Lys | NS | -0.8 | -3.9 | -3.1 | S-protein |
| 23952 | UG | Phe797Cys | NS | 2.8 | 2.5 | -0.3 | S-protein |
| 24022 | UC | Asp820Asp | S | -3.5 | -3.5 | 0 | S-protein |
| 23403 | AG | Asp614Gly | NS | -3.5 | -0.4 | 3.1 | S-protein |
| 24034 | CU | Asn824Asn | S | -3.5 | -3.5 | 0 | S-protein |
| 24325 | AG | Lys921Lys | S | -3.9 | -3.9 | -3.9 | S-protein |
| 25554 | UG | Ala54Ala | S | 1.8 | 1.8 | 0 | ORF3a |
| 25563 | GU | Glu57His | NS | -3.5 | -3.2 | 0.3 | ORF3a |
| 25775 | GU | Trp128Leu | NS | -0.9 | 3.8 | 4.7 | ORF3a |
| 25979 | GU | Gly196Val | NS | -0.4 | 4.2 | 4.6 | ORF3a |
| 26144 | GU | Gly251Val | NS | -0.4 | 4.2 | 4.6 | ORF3a |
| 26354 | UA | Leu37His | NS | 3.8 | -3.2 | -7 | E-gene |
| 26729 | UC | Ala69Ala | S | 1.8 | 1.8 | 0 | ORF5 |
| 27384 | UC | Asp183Asp | S | -3.5 | -3.5 | 0 | ORF7 |
| 28077 | GC | Val62Leu | NS | 4.2 | 3.8 | -0.4 | ORF8 |
| 28144 | UC | Leu84Ser | NS | 3.8 | -0.8 | -4.6 | ORF8 |
| 28657 | CU | Asp128Asp | S | -3.5 | -3.5 | 0 | ORF9 |
| 28854 | CU | Ser194leu | NS | -0.8 | 3.8 | 4.6 | ORF9 |
| 28863 | CU | Ser197Phe | NS | -0.8 | 2.8 | 2 | ORF9 |
| 28881 | GA | Arg203Lys | NS | -4.5 | -3.9 | 0.6 | ORF9 |
| 28882 | GA | Arg203Lys | NS | -4.5 | -3.9 | 0.6 | ORF9 |
| 28883 | GC | Gly204Arg | NS | -0.4 | -4.5 | -4.1 | ORF9 |
| 28916 | GA | Gly214Ser | NS | -4.5 | -3.9 | 0.6 | ORF9 |
| 29095 | CU | Phe274Phe | S | 2.8 | 2.8 | 0 | ORF9 |
| 29353 | CU | Tyr360Tyr | S | -1.3 | -1.3 | 0 | ORF9 |
| 29573 | GA | Val6Ile | NS | 4.2 | 4.5 | 0.3 | ORF10 |
| 29546 | CU |  | nc |  |  |  | 3'UTR |
| 29861 | GA |  | nc |  |  |  | 3'UTR |
| 29867 | UA |  | nc |  |  |  | 3'UTR |
| 29870 | CA |  | nc |  |  |  | 3'UTR |
| 29868 | GA |  | nc |  |  |  | 3'UTR |
| 29868 | GC |  | nc |  |  |  | 3'UTR |

^1^ Nucleotide coordinates are according to the SARS-CoV-2 reference genome (MN908947).

^2^ Amino acids positions are according the mature peptides in the SARS-CoV-2 reference genome (MN908947).

^3^S- synonymous (silent) mutation, NS – nonsynonymous (amino acid changing) mutation, nc – non coding.

^4^Hydrophobicity values were according to Kyte and Doolitle (1982).

^5^Reference - SARS-CoV-2 (MN908947), allele – other SARS-CoV-2 isolates.

^6^Hydrophobicity value of an amino acid in the reference sequence minus that of the allele

^7^UTR- untranslated region; ORF1ab – open reading frame with 16 non-structural proteins (nsp 1-16).
